# Effect of Reaction Time on Microwave Absorption Properties of Fe_3_O_4_ Hollow Spheres Synthesized via Ostwald Ripening

**DOI:** 10.3390/ma12182921

**Published:** 2019-09-10

**Authors:** Wei Huang, Yujiang Wang, Shicheng Wei, Bo Wang, Yi Liang, Yuwei Huang, Binshi Xu

**Affiliations:** National Key Laboratory for Remanufacturing, Army Academy of Armored Forces, Beijing 100072, China

**Keywords:** Fe_3_O_4_, hollow sphere, Ostwald ripening, attenuation ability, impedance matching, microwave absorption

## Abstract

Hollow magnetic structures have great potential to be used in the microwave absorbing field. Herein, Fe_3_O_4_ hollow spheres with different levels of hollowness were synthesized by the hydrothermal method under Ostwald ripening effect. In addition to their microstructures, the microwave absorption properties of such spheres were investigated. The results show that the grain size and hollowness of Fe_3_O_4_ hollow spheres both increase as the reaction time increases. With increasing hollowness, the attenuation ability of electromagnetic wave of Fe_3_O_4_ spheres increases first and then decreases, finally increases sharply after the spheres break down. Samples with strong attenuation ability can achieve good impedance matching, which it does preferentially as the absorber thickness increases. Fe_3_O_4_ hollow spheres show the best microwave absorption performance when the reaction time is 24 h. The minimum reflection loss (*R*_L__(min)_) can reach −40 dB, while the thickness is only 3.2 mm.

## 1. Introduction

It is well known that the structure of materials plays an important role in their thermo-mechanical behavior and transport properties. Hollow structures have shown great application prospects in lithium batteries, super capacitors, drug transport, biomedicine, gas sensors and other fields due to their advantages of large specific surface area and low density [1,2,3,4,5,6]. Template methods and template-free methods are usually used to construct hollow structures. Template-free methods usually adopt some special mechanisms, such as the Ostwald ripening effect [7], the Kendall effect [8], the current displacement effect [9], etc. Compared with template methods, template-free methods are more flexible and convenient. In recent years, with the wide application of communication technology and electromagnetic equipment in GHz, electromagnetic radiation has resulted in unprecedented pollution and seriously endangered human health [10,11,12,13]. To solve this problem, a lot of efforts have been directed towards the research and development of microwave absorbing materials (MAMs) [14,15]. As a traditional microwave absorbing material, Fe_3_O_4_ has high saturation magnetization and Curie temperature at high temperature (585 °C). This stable and excellent magnetic property has attracted many scholars’ attention. However, the high density of Fe_3_O_4_ limits its further use as a MAM [16]. 

Fortunately, many studies have shown that hollowing of Fe_3_O_4_ is an effective way to reduce material weight, produce special morphological effects and improve electromagnetic properties [17,18,19]. For example, Li et al. [20] prepared hollow Fe_3_O_4_ spheres with a diameter of 450 nm, a wall thickness of 80 nm and a density of 3.28 g/cm^3^ by solvothermal method, which was nearly 27% lower than the corresponding solid Fe_3_O_4_. Sui et al. [21] used the improved solvothermal method to control the size of Fe_3_O_4_ hollow spheres, and synthesized hollow spheres with diameters of 200–1000 nm and wall thicknesses of 35–280 nm. Xu et al. [22] prepared hollow Fe_3_O_4_ spheres with a diameter of 525 nm by thermal solvent method. When the filling mass is 60% and the thickness is 3 mm, the minimum reflection loss can reach −15.8 dB. The above research shows that the Fe_3_O_4_ hollow spheres with low density have very good application prospects in the field of microwave absorption, and that it is feasible to adjust the electromagnetic characteristics of Fe_3_O_4_ hollow spheres by morphology control. However, the relationship between the morphology of Fe_3_O_4_ hollow spheres and the microwave absorption characteristics remains to be further studied. 

In the present work, Fe_3_O_4_ hollow spheres with different levels of hollowness were prepared by Ostwald ripening mechanism. The variation of the morphology of Fe_3_O_4_ hollow spheres under the Ostwald ripening mechanism was studied. The relationships among solvothermal reaction time, structure of Fe_3_O_4_ hollow spheres and electromagnetic properties were discussed. 

## 2. Experimental

A typical preparation of hollow Fe_3_O_4_ was carried out as follows: 10 g Polyvinylpyrrolidone (PVP), 9 g FeCl_3_·6H_2_O and 36 g urea were dissolved in 400 mL ethylene glycol, and stirred for 0.5 h by electric mixer to get a deep orange transparent homogenous solution. Then, they were put into a 500 mL stainless-steel autoclave with teflon-lining, and kept at 200 °C for 8 h, 12 h, 16 h, 20 h and 24 h, respectively. After the reaction, all the reaction products were washed with water and alcohol for three times to remove the unreacted. Finally, the reaction products were collected by a magnet and dried in vacuum at 60 °C for 10 h. The code numbers of the sample at 8 h, 12 h, 16 h, 20 h and 24 h were marked as S1, S2, S3, S4 and S5, respectively.

The morphology and structure of Fe_3_O_4_ hollow microspheres were analyzed by a field emission scanning electron microscope (FESEM, JEOLJSM-6500F, Eindhoven, Holland), transmission electron microscopy (TEM, Tecnai-TF20, Oberkochen, German), and X-ray diffractometer (XRD, Japan Rigaku D/MAX-cA) using a CuKa radiation (λ = 1.5406 Å). The magnetostatic properties were characterized by vibrating sample magnetometer (VSM, BHV-55). The permeability and permittivity of samples in the frequency range 2–18 GHz were tested by a vector network analyzer (VNA, N5242A, Agilent) for simulation of reflection loss. Composite sample were realized as follows: the wax was melted at 80 °C and mixed with the Fe_3_O_4_ powder homogeneously. The mixture was moved into a toroidal mold (Φ_in_ = 3.04 mm, Φ_out_ = 7 mm). The test software (Agilent, Santa Clara, CA, USA) is 85071 and the calibration part is 85050D. Before the test, the permittivity of air was measured as an evaluation of calibration effect. 

## 3. Results and Discussion

**Figure 1:**
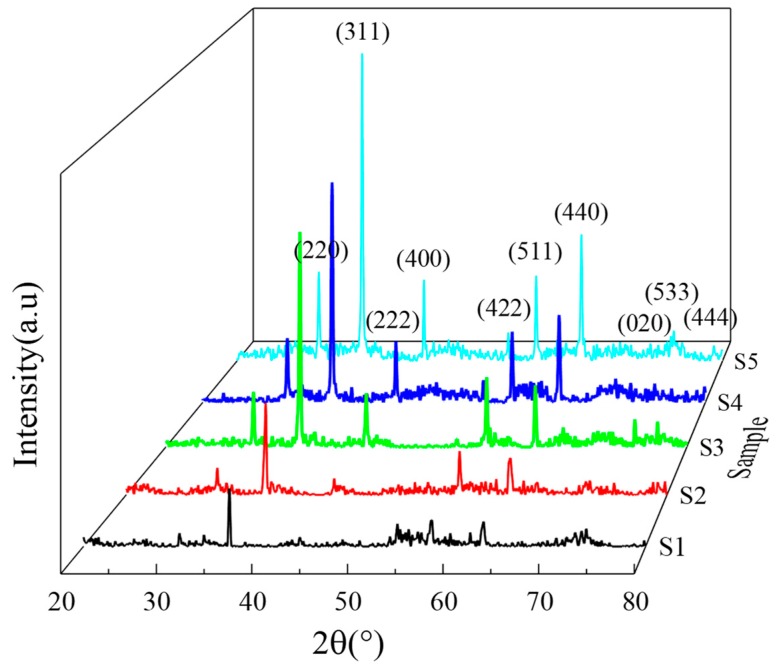
XRD patterns of Fe_3_O_4_ samples with different reaction times.

XRD results of Fe_3_O_4_ with different reaction times are shown in Figure 1. The diffraction peaks of the samples correspond well to face-centered magnetite Fe_3_O_4_ (JCPDS Card No. 99-0073), which indicates that the synthesized products have high purity. The diffraction peak intensities increase obviously as the reaction time increases. This means that the samples’ crystallinity is improved as the reaction time increases. According to the calculation results of the Scherrer formula (D = Kγ/Bcosθ), the grain size of the sample at 8 h, 12 h, 16 h, 20 h and 24 h is 25.89 nm, 30.54 nm, 38.28 nm, 41.55 nm and 48.29 nm, respectively. The results show that the grain size increases as the reaction time increases. To further identify the purity of Fe_3_O_4_, RedOx tiration (potassium dichromate) was used. Table 1 calculates n(Fe^2+^):n(Fe^3+^), Fe_σ_O_4_ (σ-nonstoichiometric) and Oxidation rate, respectively. Although Fe_3_O_4_ may be oxidized during the experiment, the results in Table 1 show that their oxidation rates are below 10%, which indicates that the purity of Fe_3_O_4_ phase in the product is very high. 

**Figure 2:**
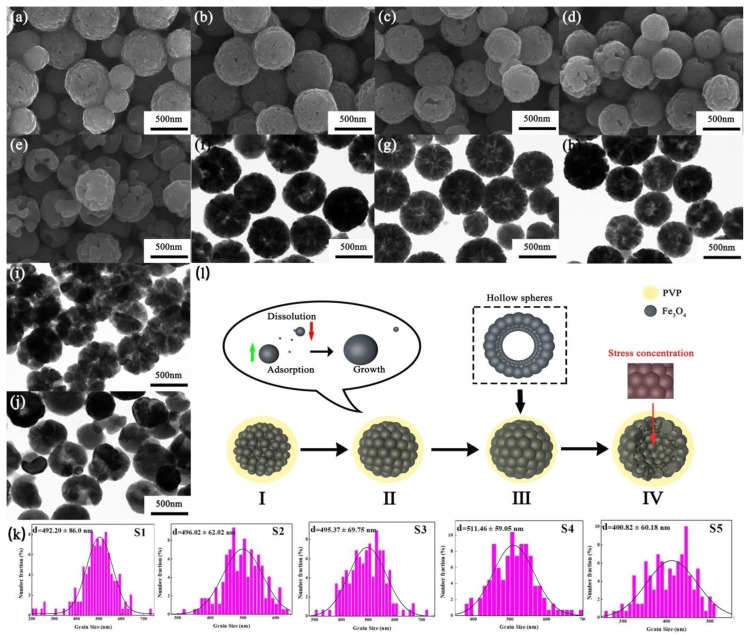
SEM of samples: (**a**) S1, (**b**) S2, (**c**) S3, (**d**) S4, (**e**) S5; TEM of samples: (**f**) S1, (**g**) S2, (**h**) S3, (**i**) S4, (**j**) S5; (**k**) Size distribution of Fe_3_O_4_ samples; (**l**) Schematic diagram of the formation process of Fe_3_O_4_ by Ostwald ripening.

To observe the morphology and internal structure of the samples, SEM and TEM analyses were carried out, and the results are illustrated in Figure 2. The prepared Fe_3_O_4_ spheres exhibit good dispersion and no agglomeration. As shown in the SEM images from Figure 2a–e, it can be seen that the surface of the Fe_3_O_4_ sphere is composed of many Fe_3_O_4_ grains, which shows the self-assembly effect of Ostwald ripening mechanism. Similar results can be seen from the TEM images, Figure 2f–j. In addition, the hollowness of Fe_3_O_4_ sphere increases gradually as the reaction time increases. The hollow size of Fe_3_O_4_ increases to breaking point as the reaction time reaches 24 h (S5). It is worth mentioning that although the formation of Fe_3_O_4_ hollow spheres reflects the typical Ostwald ripening phenomenon, the size of Fe_3_O_4_ spheres does not increase significantly during the whole reaction process. As can be seen from Figure 2k, the sizes of the Fe_3_O_4_ hollow spheres are mainly around 500 nm before they are broken. This may be due to the higher PVP concentration, which restricts the growth of Fe_3_O_4_ spheres during Ostwald ripening process [23].

Based on the results of SEM and TEM images, the formation process of Fe_3_O_4_ in this study can be explained as shown in Figure 2l. Firstly, iron ions and oxygen ions react continuously to form grains dispersed in solution under high temperature and pressure. These grains are extremely unstable due to the high surface free energy. To reduce the surface free energy, they will be aggregated into larger loose microspheres, which are the embryonic form of Fe_3_O_4_ spheres, as illustrated in Figure 2l-I. From Figure 2l-II to III, with the prolongation of reaction time, the primary grains in outer layer of the microspheres contact with the solution sufficiently, and the absorption rate of iron and oxygen ions from the solution is faster, resulting in the larger grain size than the internal grain size. Then, the internal grains dissolve into the surrounding medium gradually, and re-precipitate on the surface of the external grains due to the higher surface free energy of internal grains. Therefore, the external grains grow further, leading to the formation of Fe_3_O_4_ spheres with hollow structure. In addition, there is residual stress in the external grains due to the continuous growth of Fe_3_O_4_ grains and the restraint of high PVP concentration. Excessive residual stress leads to the cracks at grain boundaries, resulting in crystal rupture, and the rupture of Fe_3_O_4_ spheres ultimately, as shown in Figure 2l-IV. 

**Figure 3:**
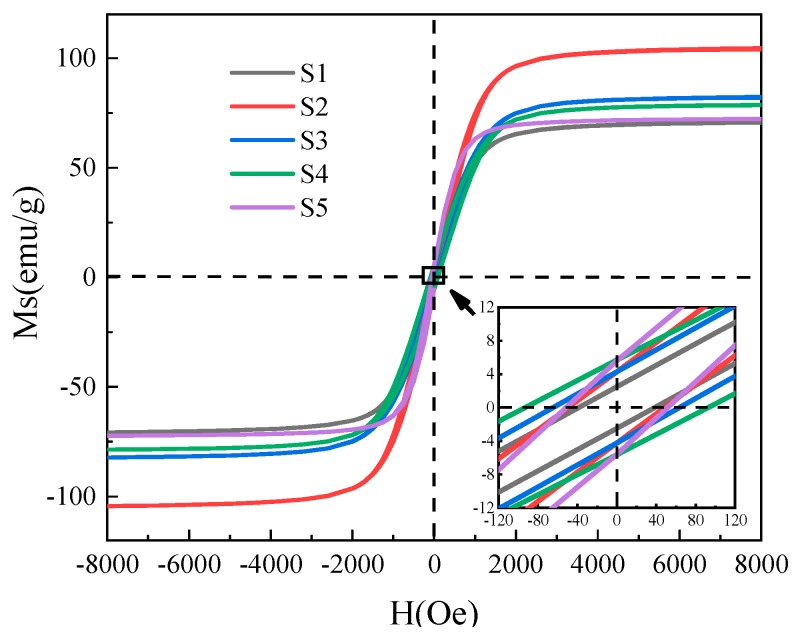
Magnetic hysteresis loop of Fe_3_O_4_ hollow spheres at room temperature.

Figure 3 is a comparison diagram of hysteresis loops for Fe_3_O_4_ samples at different reaction time. The saturation magnetization and coercivity of Fe_3_O_4_ samples are shown in Table 2. The morphology of Fe_3_O_4_ has a significant effect on its magnetic properties. As the reaction time is prolonged, the defects of the sample decrease, the crystallinity is improved, and the super-exchange effect of the Fe–O–Fe bond is enhanced, resulting in a higher saturation magnetization [24]. However, the saturation magnetization of Fe_3_O_4_ is woken up as the reaction time is in the range of 12–24 h, which may be due to the hollow structure reducing the magnetic coupling between the Fe_3_O_4_ grains. The hollow structure may have a certain strengthening effect on the coercive force of Fe_3_O_4_, which is manifested in the increase in the coercive force of the Fe_3_O_4_ sphere at higher hollowness, and which suddenly decreases when the hollow sphere is broken. The report [25] shows that the critical size of superparamagnetism of Fe_3_O_4_ nanoparticles is 25 nm. Fe_3_O_4_ grains prepared in this paper are generally larger than this value, thus showing so it shows ferromagnetism. 

**Figure 4:**
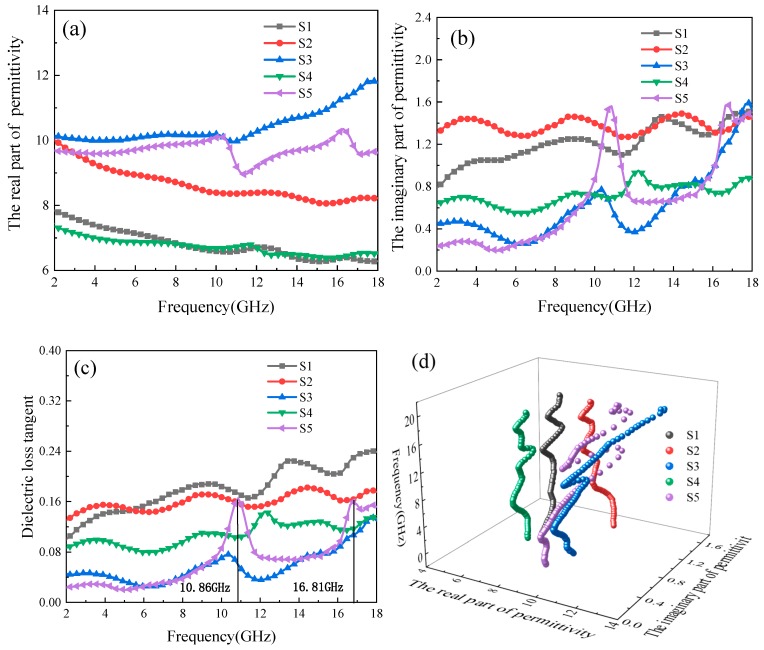
(**a**) The real part of permittivity, (**b**) the imaginary part of permittivity, (**c**) dielectric loss tangent, and (**d**) Cole-Cole curves of Fe_3_O_4_ in the range of 2–18 GHz.

The frequency dependence of complex permittivity (*ε*_r_ = *ε*′ − j*ε*″), dielectric loss tangent and Cole-Cole semicircle of samples in the range of 2–18 GHz are illustrated in Figure 4. Generally speaking, the real part of permittivity ε′ is produced by various displacement polarization within the material, it represents the energy storage term of the material. The imaginary part of permittivity, ε″, is produced by various relaxation polarizations caused by steering polarization in the material, it cannot keep up with the change of external high frequency electric field, and represents the loss term of material. As shown in Figure 4a,b, the complex permittivity of Fe_3_O_4_ hollow spheres with different morphologies varies significantly with the increase of reaction time. The variation ranges of real and imaginary parts of complex permittivity are 6.1–11.8 and 0.2–1.5, respectively. The dielectric properties of different samples vary greatly and show no obvious regularity, which is related to the complex variation of polarization and conductivity affected by the grain size of Fe_3_O_4_ and the state of dispersion and aggregation. 

The dielectric loss tangent (tan *δ*_E_ = *ε*″/*ε*′) reflects the dielectric loss ability of material. Tan *δ*_E_ of Fe_3_O_4_ samples at different time are shown in Figure 4c. ε′ changes gently with frequency; the trend of tan *δ*_E_ is similar to ε″. Tan *δ*_E_ decreases with the prolongation of reaction time (S1–S3) before the rupture of Fe_3_O_4_ spheres, and increases slightly as the reaction time reaches 20 h (S4). There are two obvious loss peaks in tan *δ*_E_ curve with frequency after the rupture of Fe_3_O_4_ spheres (S5), at 10.86 GHz and 16.82 GHz, respectively. The appearance of the loss peak increases the dielectric loss capacity of S5 at this frequency greatly. According to Debye relaxation theory, the real and imaginary parts of complex permittivity can be transformed into the following form [26]:

(1) $$\varepsilon_{r}=\varepsilon_{\infty }+\frac{\varepsilon_{s}-\varepsilon_{\infty }}{1+j2\pi f\tau }=\varepsilon^{\prime }-j\varepsilon^{\prime \prime }$$

In Formula (1), *f* is the frequency, *ε*_∞_ and *ε*_s_ are relative dielectric permittivity at the high-frequency limit and static dielectric permittivity, respectively, and τ is the relaxation time. Therefore, *ε*′ and *ε*″ can be further transformed:

(2) $$\varepsilon^{\prime }=\varepsilon_{\infty }+\frac{\varepsilon_{s}-\varepsilon_{\infty }}{1+{\left(2\pi f\right)}^{2}\tau^{2}}$$

(3) $$\varepsilon^{\prime \prime }=\frac{2\pi f\tau \left(\varepsilon_{s}-\varepsilon_{\infty }\right)}{1+{\left(2\pi f\right)}^{2}\tau^{2}}$$

According to Formulas (2) and (3), the relationship between *ε*′ and *ε*″ can be further deduced:

(4) $${\left(\varepsilon^{\prime }-\frac{\varepsilon_{s}+\varepsilon_{\infty }}{2}\right)}^{2}+{\left(\varepsilon^{\prime \prime }\right)}^{2}={\left(\frac{\varepsilon_{s}-\varepsilon_{\infty }}{2}\right)}^{2}$$

According to Formula (4). If the polarization of the permittivity is caused by Debye relaxation process, it will be shown as a Cole-Cole semicircle. Based on the above theory, 3D illustration of Cole-Cole curves is made as shown in Figure 4d. First, for S1–S4, no Debye semicircles exist, and the Cole-Cole curves are just twisted spirals. Even for S5, it is not an ideal Debye semicircle. According to Formulas (2) and (3), if the dielectric spectrum satisfies the Debye relaxation equation, the maximum value of ε″ appears and *ε*″ = (*ε*_s_ − *ε*_∞_)/2 when 2π*f* = *τ*^−1^. However, the *ε*′ of S5 is maintained at about 10 initially, then decreases to 8.9 from 10.3 to 11.4 GHz, resulting in a value that corresponds to a loss peak of ε″ (10.86 GHz). Simply estimated, the maximum value of the loss peak is much larger than that of (*ε*_s_ − *ε*_∞_)/2, indicating that the dielectric behavior is not a typical Debye relaxation. This behavior may correspond to a resonance process similar to dielectric relaxation. Overall, we consider that the dielectric loss of Fe_3_O_4_ is not Debye relaxation, but resonant dielectric response.

**Figure 5:**
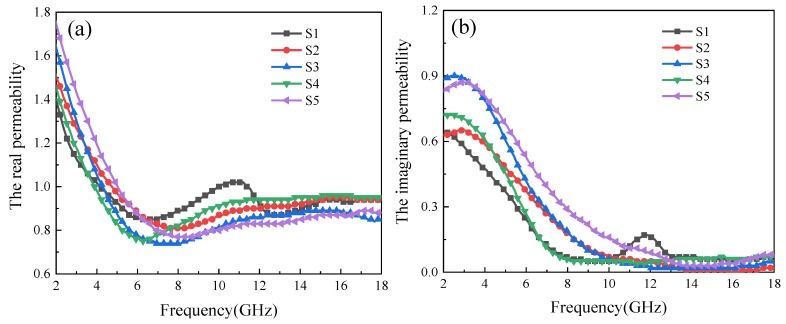

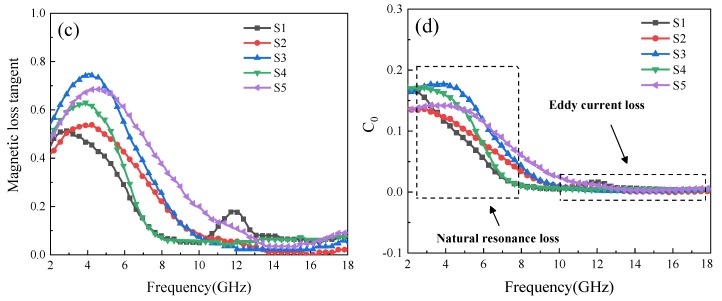
(**a**) The real part of permeability, (**b**) the imaginary part of permeability, (**c**) magnetic loss tangent, and (**d**) C_0_ curves in the range of 2–18 GHz.

Figure 5 displays the frequency dependence of complex permeability (*μ*_r_ = *μ*′ − *jμ*″), magnetic loss tangent and *C*_0_ of samples in the range of 2–18 GHz. From Figure 5a,b, it can be seen that real part of permeability (*μ*′) and imaginary part of permeability (*μ*″) curves exhibit a similar trend, decreasing first and then remaining stable. This indicates that the magnetic loss ability of Fe_3_O_4_ is mainly reflected in the S band (2–4 GHz) and C band (4–8 GHz), and Ostwald ripening has less effect on the magnetic properties than on the dielectric properties of Fe_3_O_4_. Similarly to dielectric loss, the magnetic loss tangent (tan *δ*_M_ = *μ*″/*μ*′) reflects the magnetic loss ability of materials, and tan *δ*_M_ can also be observed in Figure 5c. The results show that there is a loss peak at the tested frequency of tan *δ*_M_, at which is much larger than that of tan *δ*_E_. Tan *δ*_M_ decreases slowly and finally approaches to tan *δ*_E_ as the frequency increases. Therefore, it can be judged that the loss mechanism of Fe_3_O_4_ is mainly magnetic loss. For ferrite materials, magnetic loss is usually caused by hysteresis loss, eddy current loss, domain wall displacement and natural resonance. However, hysteresis loss can be neglected in the case of weak applied electric field, and domain wall displacement only occurs in the MHz range, so the magnetic loss mechanism in Fe_3_O_4_ is generally related to eddy current loss and natural resonance. Material thickness (*d*) and conductivity (*σ*) are two main factors affecting eddy current loss, which can be expressed as follows [27]:

(5) $$C_{0}=\mu^{\prime \prime }{\left(\mu^{\prime }\right)}^{-2}f^{-1}=\frac{2}{3}\pi \mu_{0}d^{2}\sigma$$

If the magnetic loss is caused entirely by the eddy current loss, the right side of Equation (5) is constant, so no the variation of *C*_0_ with frequency should be revealed in a constant. In other words, the eddy current loss should be dominant in the frequency range of relatively stable and small fluctuations. As shown in Figure 5d, *C*_0_ curves of Fe_3_O_4_ fluctuate greatly at low frequencies and tend to be stable at the middle and high frequencies, indicating that the magnetic loss should be caused by natural resonance at low frequency, and as the frequency increases, it gradually changes into eddy current loss. Combined with Figure 5c, the corresponding frequencies of loss peaks caused by natural resonance at low frequencies are 2.86 GHz (S1), 4.12 GHz (S2), 4.16 GHz (S3), 3.77 GHz (S4) and 4.55 GHz (S5), respectively. The difference of natural resonance frequencies between different samples is caused by the effective anisotropy field of magneto-crystals with different morphologies of Fe_3_O_4_ [28]. In addition, *C*_0_ approaches zero at the middle and high frequencies, indicating that the eddy current loss is very weak. This is consistent with the weak magnetic loss at the corresponding frequency in Figure 5c. It is noteworthy that there is also a loss peak at 12 GHz in S1, which is caused by exchange resonance. A relevant report shows that there is exchange resonance as the grain size of Fe_3_O_4_ is about 10 nm [29]. In addition, S1 is the early formation stage of the Fe_3_O_4_ hollow spheres; there are some grains about 10 nm in size, so the speculation of exchange resonance in S1 is valid.

The energy of the electromagnetic wave is transferred to heat energy by dielectric loss and magnetic loss as the electromagnetic wave enters into the absorber. The attenuation constant α determines the capability attenuation characteristics of materials to attenuate the electromagnetic wave, which can be expressed by the following formula [30,31,32];

(6) $$\alpha =\frac{\sqrt{2}\pi f}{c}\sqrt{\mu^{\prime \prime }\varepsilon^{\prime \prime }-\mu^{\prime }\varepsilon^{\prime }+\sqrt{{\left(\mu^{\prime \prime }\varepsilon^{\prime \prime }-\mu^{\prime }\varepsilon^{\prime }\right)}^{2}+{\left(\varepsilon^{\prime }\mu^{\prime \prime }+\varepsilon^{\prime \prime }\mu^{\prime }\right)}^{2}}}$$

**Figure 6:**
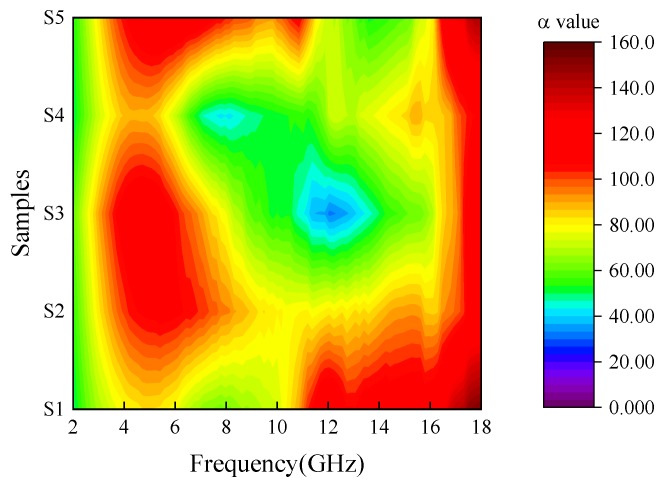
Attenuation coefficient contour map of Fe_3_O_4_ samples in the range of 2–18 GHz.

In Formula (6), *c* and *f* represent the velocity of light and frequency, respectively. The attenuation coefficient α of different Fe_3_O_4_ samples in the frequency range of 2–18 GHz can be visually expressed by the contour plot map, as shown in Figure 6. The result shows that the attenuation ability of Fe_3_O_4_ to electromagnetic wave is mainly reflected in the low and high frequency bands, while the attenuation ability of Fe_3_O_4_ to the medium frequency band is weak. With increasing reaction time, the attenuation coefficient of the intermediate frequency band decreases gradually, reaches the minimum at 20 h (S4), and increases after the sphere ruptures at 24 h (S5). To study the attenuation ability of different samples in each work frequency bands, the integral values of α in each band are calculated. The specific results are shown in Table 3. When the reaction time is 8–16 h (S1–S3), the total integral value of α in the range of 2–18 GHz increases, then decreases sharply (S4). When the sphere ruptures, the total integral value rises immediately. Based on the above calculation results, the electromagnetic wave absorption capacity of the samples can be arranged in the following order: S5 > S3 > S2 > S1 > S4. 

On the basis of the complex permittivity and complex permeability data, the reflection loss (*R*_L_) of Fe_3_O_4_ can be deduced from the transmission line theory [33]:

(7) $$R_{L}=20lg\left|\frac{Z_{\mathrm{in}}-Z_{0}}{Z_{\mathrm{in}}+Z_{0}}\right|$$

(8) $$Z_{\mathrm{in}}=\sqrt{\frac{\mu_{r}}{\varepsilon_{r}}}\tanh\left|j\frac{2\pi df}{c}\sqrt{\mu_{r}\varepsilon_{r}}\right|$$

**Figure 7:**
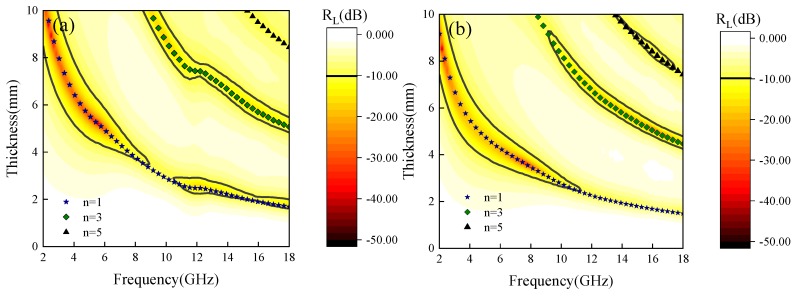

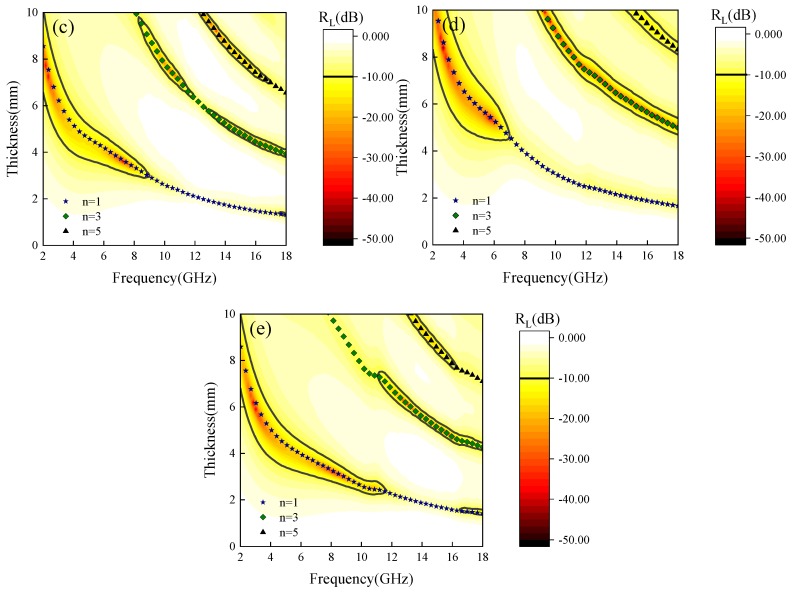
Fe_3_O_4_ reflection loss diagrams: (**a**) S1, (**b**) S2, (**c**) S3, (**d**) S4 and (**e**) S5.

Among them, *Z*_in_ stands for the input impedance of absorbing material, *Z*_0_ is the impedance of free space, *c* is the speed of light in vacuum, *d* is the thickness of absorber, and *f* represents microwave frequency. Based on the above formulas, *R*_L_ can be simulated as the thickness is in the range of 0–10 mm and the frequency is 2–18 GHz, as depicted in Figure 7a–e. The absorption peak of the sample moves to low frequency gradually as the thickness increases, which can be explained by the equation [34]: *f = c/2πdμ″*, where *f* represents the optimal matching frequency, *d* is the optimal matching thickness. Therefore, the absorption band can be adjusted by changing the thickness of Fe_3_O_4_ to meet the actual needs. Moreover, when the thickness of the sample exceeds a certain value (S1—4.8 mm, S2—4.3 mm, S3—3.8 mm, S4—4.8 mm, S5—4.2 mm), there are absorption peaks at both low and high frequencies, which indicates that Fe_3_O_4_ is promising as a low frequency and high frequency compatible microwave absorbing material. In addition, *R*_L_ is very sensitive to the change of thickness and can produce very strong reflection loss at a specific thickness. That can be explained by a quarter-wavelength model [35]:

(9) $$t_{m}=\frac{nc}{4f_{m}\sqrt{\left|\varepsilon_{r}\right|\left|\mu_{r}\right|}};\;n\;=\;1,\;3,\;5,\;\ldots$$

Where |*ε*_r_| and |*μ*_r_| are the modulus of *ε*_r_ and *μ*_r_ at *f*_m_, respectively. If the thickness of the absorber is equal to the calculated *t*_m_, the interference effect will occur, and the electromagnetic wave will be attenuated greatly. When n = 1, 3 and 5, the frequency dependence of *t*_m_ is calculated and plotted on the contour maps of S1–S5 in Figure 7. It can be observed that almost all the points of *R*_L (min)_ on the curves of *t*_m_. Thus, we can judge that the absorption peaks of Fe_3_O_4_ samples are aroused by thickness resonance to the specific frequency microwave.

**Figure 8:**
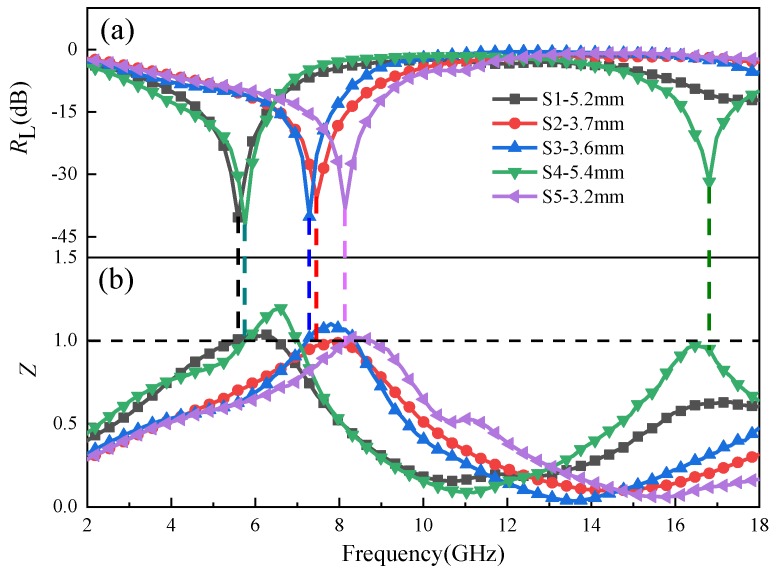
(**a**) The *R*_L_ and (**b**) *Z* of Fe_3_O_4_ samples at resonance thickness.

As the thickness of the sample increases, the resonance thicknesses are satisfied, with the quarter-wavelength model appearing in turn, which are 3.2 mm—S5, 3.6 mm—S3, 3.7 mm—S2, 5.2 mm—S1 and 5.4 mm—S4, respectively. The order is consistent with the order of attenuation ability mentioned above and the *R*_L_ at resonance thickness are shown in Figure 8a. The absorption band of samples at the resonance thickness is mainly concentrated at low frequency, it might be related to the strong natural resonance loss at low frequency. Additionally, *R*_L (min)_ of each sample can approach −40 dB, which is equivalent to 99.99% of the electromagnetic wave energy absorbed, indicating that Fe_3_O_4_ possesses great potential as an excellent low-frequency microwave absorption material. The normalized characteristic impedance Z is a key parameter in reducing the reflection of the electromagnetic wave, which can be expressed by the following equation [36]:

(10) $$Z=\frac{Z_{\mathrm{in}}}{Z_{0}}=\sqrt{\frac{\mu_{r}}{\varepsilon_{r}}}\tanh\left|j\frac{2\pi df}{c}\sqrt{\mu_{r}\varepsilon_{r}}\right|$$

Input impedance *Z*_in_ should be equal to the free space impedance *Z*_0_, so that the electromagnetic wave can enter into the absorber completely and can be totally attenuated completely; thus, Z should be as close as possible to 1. As shown in Figure 8b, at the optimum thickness and frequency of the sample, the Z values corresponding to the loss peaks are closed to 1, indicating that they are well matched with the free space impedance. In addition, by comparing the attenuation ability, the relationship between resonance thickness and Z, it is easy to draw the conclusion that the impedance matching of Fe_3_O_4_ hollow spheres can be achieved in a thinner case when the attenuation ability is strong.

## 4. Conclusions

Fe_3_O_4_ hollow spheres were fabricated with different reaction times by Ostwald ripening process. The microstructure and electromagnetic properties have been investigated systemically. Fe_3_O_4_ spheres all have hollow structure at different reaction times ranging from 8 h to 24 h, the size of Fe_3_O_4_ spheres remains at about 500 nm in diameter. The grain size and hollowness of Fe_3_O_4_ spheres increase as the reaction time. Fe_3_O_4_ hollow spheres break down as the reaction time reaches 24 h. With increasing in hollowness, the electromagnetic wave attenuation ability of Fe_3_O_4_ spheres increases first and then decreases, and increases sharply after the spheres finally break down. There is an obvious effect on the dielectric and magnetic properties of Fe_3_O_4_ spheres with different morphologies, resulting in a significant difference in the attenuation ability of different samples to electromagnetic waves. The electromagnetic attenuation ability of Fe_3_O_4_ spheres is S5 > S3 > S2 > S1 > S4. Sample with strong attenuation ability can achieve impedance matching preferentially with an increase in thickness. In addition, Fe_3_O_4_ hollow nanospheres exhibit good microwave absorption properties due to the strong natural resonance loss and interference cancellation of electromagnetic wave. *R*_L (min)_ can reach −40 dB as the thickness is only 3.2 mm and the reaction time is 24 h.

## Figures and Tables

**Table 1:** Purity analysis of Fe_3_O_4_ by RedOx tiration: n(Fe^2+^):n(Fe^3^^+^), Fe_σ_O_4_ (σ-nonstoichiometric) and oxidation rate.

Sample	n(Fe^2+^):n(Fe^3+^)	Fe_σ_O_4_	Oxidation Rate
S1	0.449	Fe_2.974_O_4_	7.21 %
S2	0.463	Fe_2.982_O_4_	5.27 %
S3	0.455	Fe_2.977_O_4_	6.38 %
S4	0.459	Fe_2.979_O_4_	5.82 %
S5	0.453	Fe_2.976_O_4_	6.65 %

**Table 2:** Magnetic properties of Fe_3_O_4_ hollow spheres at different reaction time.

Sample	Saturation Magnetization (emu/g)	Coercive Force (H/Oe)
S1	70.64	74.7
S2	103.93	98.8
S3	81.83	129.7
S4	78.55	188.8
S5	72.27	101.4

**Table 3:** Integral values of attenuation coefficients of Fe_3_O_4_ samples at different bands.

Sample	S Band	C Band	X Band	Ku Band	Total
S1	90.09	125.41	131.85	383.46	730.81
S2	101.57	214.96	122.20	459.66	898.39
S3	182.44	382.66	90.10	245.88	901.08
S4	111.83	173.48	99.50	201.79	586.60
S5	154.03	415.93	178.51	184.52	932.99
